# Anthropometric and Biochemical Correlations of Insulin Resistance in a Middle-Aged Maltese Caucasian Population

**DOI:** 10.1155/2024/5528250

**Published:** 2024-02-21

**Authors:** Rachel Agius, Nikolai Paul Pace, Stephen Fava

**Affiliations:** ^1^Faculty of Medicine and Surgery, University of Malta, Tal-Qroqq, Msida, Malta; ^2^Mater Dei Hospital, Triq Dun Karm, Msida MSD2090, Malta

## Abstract

**Background:**

Insulin resistance (IR) is associated with increased cardiovascular disease risk, and with increased all-cause, cardiovascular, and cancer mortality. A number of surrogate markers are used in clinical practice to diagnose IR. The aim of this study was to investigate the discriminatory power of a number of routinely available anthropometric and biochemical variables in predicting IR and to determine their optimal cutoffs.

**Methods:**

We performed a cross-sectional study in a cohort of middle-aged individuals. We used receiver operator characteristics (ROC) analyses in order to determine the discriminatory power of parameters of interest in detecting IR, which was defined as homeostatic model assessment-insulin resistance ≥2.5.

**Results:**

Both the lipid accumulation product (LAP) and visceral adiposity index (VAI) exhibited good discriminatory power to detect IR in both males and females. The optimal cutoffs were 42.5 and 1.44, respectively, in males and 36.2 and 1.41, respectively, in females. Serum triglycerides (TG) and waist circumference (WC) similarly demonstrated good discriminatory power in detecting IR in both sexes. The optimal cutoffs for serum TG and WC were 1.35 mmol/L and 96.5 cm, respectively, in men and 1.33 mmol/L and 82 cm, respectively, in women. On the other hand, systolic and diastolic blood pressure, liver transaminases, high-density lipoprotein cholesterol, serum uric acid, ferritin, waist-hip ratio, “A” body shape, thigh circumference, and weight-adjusted thigh circumference all had poor discriminatory power.

**Conclusions:**

Our data show that LAP, VAI, TG, and WC all have good discriminatory power in detecting IR in both men and women. The optimal cutoffs for TG and WC were lower than those currently recommended in both sexes. Replication studies are required in different subpopulations and different ethnicities in order to be able to update the current cut points to ones which reflect the contemporary population as well as to evaluate their longitudinal relationship with longer-term cardiometabolic outcomes.

## 1. Introduction

Hyperinsulinaemia and insulin resistance (IR) are associated with increased cardiovascular disease risk [1–3], as well as with increased cardiovascular, cancer, and all-cause mortality [4, 5]. The dysfunction associated with insulin resistance is largely restricted to the phosphatidylinositol 3-kinase pathway rather than the mitogen-activated protein kinase (MAPK) pathway. The former mediates the anabolic effects of insulin, whilst the latter mediates the mitogenic and proinflammatory effects of [6]. The hyperinsulinaemia associated with IR therefore fuels increased MAPK pathway activity. The resultant enhanced mitogenecity probably mediates the increased cancer risk associated with IR. The chronic subclinical proinflammatory state drives endothelial dysfunction [7], which in turn predisposes to atherosclerosis and to hypertension. The dyslipidaemia typically associated with IR, namely, decreased and dysfunctional high-density lipoprotein (HDL), increased very low-density lipoprotein, and the generation of small dense and oxidized low-density lipoprotein (LDL), also contributes to the increased cardiovascular risk. Furthermore, oxidized LDL may also increase the risk of certain cancers [8].

However, routine quantification of IR is not readily available in clinical practice. The euglycemic insulin clamp is the gold standard for measuring IR, whereby subjects are given continuous insulin infusion with plasma glucose levels being maintained constant by varying the rate of glucose infusion. The glucose infusion rate is therefore a measure of insulin sensitivity [9]. Whilst being a valuable research tool, this is clearly impractical for use in a clinical setting. A number of surrogate markers of IR have therefore been devised. These include the Homeostatic Model Assessment of Insulin Resistance (HOMA-IR) [10] and Quantitative Insulin Sensitivity Check Index (QUICKI) [11]. However, both require the measurement of fasting serum insulin which is often not available in routine clinical care.

A number of anthropometric and biochemical parameters are therefore used as surrogate indices of IR. However, data comparing the discriminatory power of these parameters are lacking. Furthermore, the cutoffs for each parameter is uncertain, with different bodies using different cutoffs. It is also important to note that these cutoffs were developed around 20 years ago. Many secular changes might have contributed to change in the optimal cut-offs of the various parameters to predict IR. These include changes in dietary habits [12, 13], an increase in adiposity [14–16], changes in body fat distribution patterns [16, 17], and a decrease in muscle mass [18, 19]. Additionally, anthropometric-based indices of IR require cross-population replication and validation to account for regional differences in body composition and obesity prevalence which are partly determined by population genetic structure.

The aim of the present study was to determine the discriminatory power of the various anthropometric and biochemical parameters in predicting IR and to determine the optimal cutoffs using receiver operating characteristic (ROC) analysis in a contemporary population. IR was defined as HOMA-IR ≥ 2.5. We used this cutoff since it has been shown to predict increased mortality in large population-based studies [20, 21]. In view of sex differences in the relationship of anthropometric and biochemical parameters with IR [22], we investigated males and females separately.

## 2. Methods

**Figure 1:**
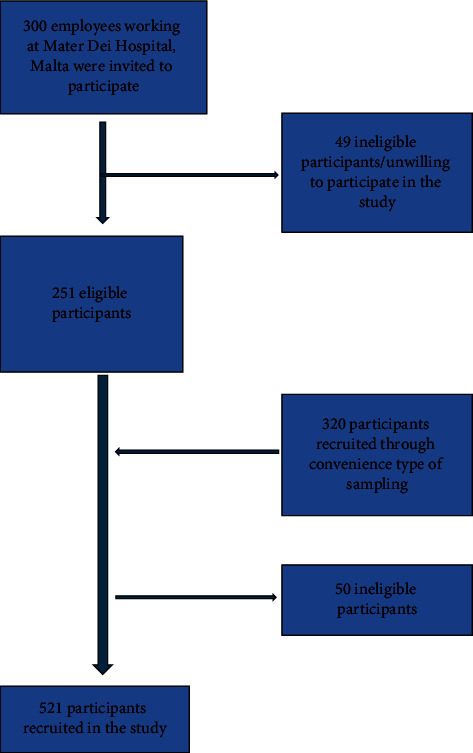
Flowchart of participant recruitment.

This was a cross-sectional study consisting of 521 middle-aged, (41 ± 10 years) non-institutionalized individuals of Maltese Caucasian descent. The sample population was identified after a letter of invitation (recruitment letter) was sent either electronically (via email) or via post to individuals who fit the eligibility criteria for this survey. Initially, invites were sent to employees who worked at the Mater Dei Teaching Hospital in Malta, and subsequently, a convenience type of sampling was carried out whereby the recruited individual (index person) was allowed to invite other colleagues/friends/family members as new participants in the study through word of mouth or by passing on the recruitment letter via email or post. This method of recruitment was similar to that used by Buscemi et al. in the ABCD study [23]. The exclusion criteria were active malignancy or terminal illness, type 1 diabetes, pregnancy, genetic or endocrine causes of overweight or underweight (apart from controlled thyroid disorders), and inability to give voluntary informed consent.  Figure 1 shows the flowchart of participant recruitment. A dedicated questionnaire was used to capture baseline demographic data relating to age, sex, past medical and surgical history, and a detailed drug history including use of antihypertensives, and hypolipemic agents.

Anthropometric measurements were taken with the participants dressed in light clothing and without shoes, using validated equipment which was calibrated in accordance with WHO recommendations [24]. Body weight was measured to the nearest 0.1 kg, whilst height and all circumferences to the nearest 0.1 cm. Height was measured using a calibrated stadiometer. Body mass index (BMI) was calculated as the weight (in kg) divided by the square of the height (in meters). Waist circumference (WC) was measured over the abdomen halfway between the bottom of the rib cage and superior iliac crest; the hip circumference (HC) was measured over the widest diameter around the buttocks with participants standing with their feet together and after full expiration. The neck circumference (NC) was measured at level of the mid-cervical spine [25]. The mid-upper arm circumference (MUAC) was measured at the midpoint of the distance between the acromion and olecranon process, with the elbow flexed at a 90° and the arm held parallel to the side of the body. The thigh circumference (ThC) was measured at the level of the gluteal fold with the thigh muscles fully relaxed. All circumferences were taken with the subjects standing upright, with shoulders and thighs relaxed, facing the investigator [26]. Waist-to-height ratio (WHtR), waist-to-hip ratio (WHR), waist-to-thigh ratio, and arm-to-height ratio were calculated as WC (cm)/height (cm), WC (cm)/HC (cm), WC (cm)/ThC (cm), and MUAC/height, respectively. The conicity index (CI) was calculated using the formula CI = waist/(0.109 × √weight (kg)/height (m) [27], the body adiposity index (BAI) was calculated using the formula BAI = (HC/height^3/2^) − 18 [28], abdominal volume index (AVI) was measured according to the formula AVI = (2 cm (waist)^2^ + 0.7 cm (waist-hip)^2^)/1000 [29], whilst A-type body shape index (ABSI) was measured using the formula ABSI = WC/BMI^2/3^ × height^½^ [30]. Blood pressure was measured after 5 minute rest in the seated position using a clinically validated digital sphygmomanometer with an appropriately sized cuff for each participant and using the average of the second and third readings for analyses, in accordance with the European Society of Hypertension Guidelines [31].

Fasting plasma glucose (FPG), HbA_1c_, lipid, and other biochemical parameters including renal profile, liver function tests, and thyroid function tests were assayed. All investigations were performed at the Biochemistry Laboratory of Mater Dei Hospital, Malta using automated and regularly calibrated analysers. FPG and lipid profile assays were performed using COBAS INTEGRA® Analysers (Roche diagnostics, Basel, Switzerland) machines. Fluoride-containing tubes were used for the collection of samples for the estimation of FPG so as to reduce glycolysis. The FPG assay was based on hexokinase and glucose oxidase enzyme reactions. Blood for the lipid profile assessment was collected in a serum clot activator tube. The lipid variables measured were total cholesterol, HDL-cholesterol (HDL-C), LDL-cholesterol (LDL-C), and triglycerides (TG). Haemoglobin A_1c_ was measured using the Bio-Rad variant II HbA_1c_ program (California, USA), which utilises the principle of high pressure liquid chromatography. Insulin was quantified by sandwich ELISA (Diagnostic Automation, USA) using a Mithras® microplate reader for absorbance determination as per the manufacturer's instructions. Samples were assayed in duplicate using 50 *μ*L of serum HOMA-IR was calculated using the formula: fasting serum insulin (*μ*U/ml) × fasting plasma glucose (mmol/L)/22.5 [10].

The lipid accumulation product (LAP) was calculated by the formulae LAP = (WC-65) × (TG) in males, and (WC-58) × (TG) in females (WC in cm and TG in mmol/l) [32]. The atherogenic index of plasma (AIP) was calculated as per the formula of Hermans et al. (log(TG/HDL-C)) [33], whilst body roundness index (BRI) was described as instructed by Thomas et al. [34]. The visceral adiposity index (VAI) was calculated as VAI = (WC (cm)/(39.68 + (1.88^*∗*^BMI) × (TG/1.03) × (1.31/HDL-C) for men and VAI = (WC(cm)/(36.58 + (BMI × 1.89) × (TG/0.81) × (1.52/HDL-C) for women [35].

All participants gave their written informed consent stating willingness to participate in this study as well as to undergo physical examination and biochemical testing. Ethical and data protection approvals were granted from the University of Malta Research Ethics Committee (Ref No: 06/2016) of the Faculty of Medicine and Surgery and the Information and Data Protection Commissioner respectively.

### 2.1. Statistical Methods

Normality of continuous variables was assessed by the Shapiro–Wilk and Kolmogorov–Smirnov tests. All continuous parameters exhibited a skewed nonnormal distribution, and nonparametric statistics with medians and interquartile ranges are presented. The statistical significance of differences in proportions was assessed using the two proportions *z* test. Spearman's rank-order coefficient was used to explore the strength and direction of association between quantitative variables.

Receiver operating characteristic (ROC) analysis was used to compute the area under curve (AUC) to assess the performance of anthropometric and biochemical parameters, and indices derived thereof, in discriminating subjects with insulin resistance (defined by the categorical cutoff HOMA-IR ≥ 2.5). The highest Youden index (sensitivity + specificity − 1) was used to determine optimal cutoff points. ROC analysis was performed using the easyROC R application [36], and cutoff values were determined using the OptimalCutpoints R package [37]. All analyses were performed using IBM SPSS version 26 and R v.3.4.2. A *p* value of <0.05 was considered significant.

## 3. Results

**Figure 2:**
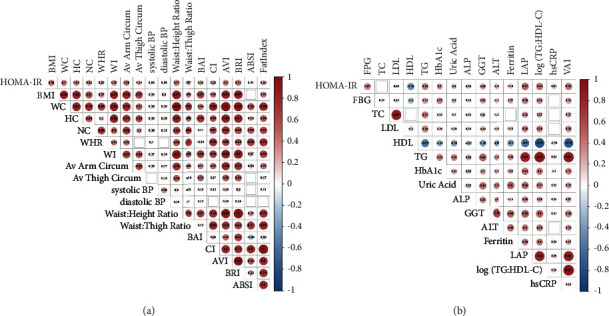
Correlation matrix between HOMA-IR level and anthropometric/clinical indices of adiposity (a) and biochemical parameters (b). Colour depicts Spearman's rank order correlation coefficient, with circle size and colour intensity indicating the magnitude of the correlation coefficient. Significant correlation coefficients are labelled; empty cells represent insignificant correlation between indices. HOMA-IR = homeostatic model assessment of insulin resistance; BMI = body mass index; WC = waist circumference; HC = hip circumference; NC = neck circumference; WHR = waist-hip ratio; WI = waist index; BAI = body adiposity index; CI = conicity index; BRI = body roundedness index; AVI = abdominal volume index; FPG = fasting plasma glucose; TC = total cholesterol; LDL = low-density lipoprotein cholesterol; HDL = high-density lipoprotein cholesterol; TG = triglycerides; HbA1c = glycated haemoglobin; ALP = alkaline phosphatase; GGT = gamma glutamyl transferase; ALT = alanine transaminase; LAP = lipid accumulation product; hsCRP = high-sensitivity C-reactive protein; and VAI = visceral adiposity index.

Five hundred and twenty-one subjects participated in the study (331 females and 190 males). The median (interquartile range) age was 41 (6.0) years.  Table 1 shows subject characteristics stratified by sex and HOMA-IR ≥ 2.5. As expected, subjects with HOMA-IR ≥ 2.5 had higher BMI, WC, FPG, and TG but a lower HDL-C. There was also a higher proportion of use of antihypertensive medication in both males and females with HOMA-IR ≥ 2.5 and of lipid-lowering pharmacotherapy in females with HOMA-IR ≥ 2.5.  Figure 2 shows a correlation matrix of HOMA-IR with quantitative anthropometric and biochemical indices. As expected, there were significant positive correlations between HOMA-IR and anthropometric or biochemical indices of adiposity.

**Figure 3:**
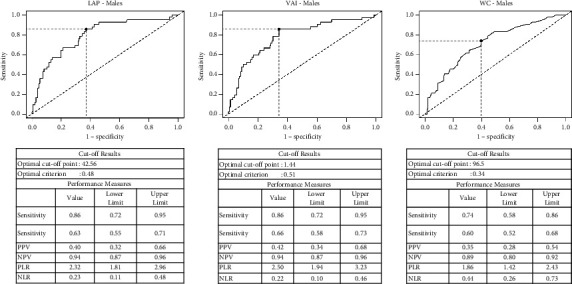
Receiver operator characteristic curves for lipid accumulation index, visceral adiposity index, and waist circumference to predict insulin resistance (HOMA-IR > 2.5) in males.

In males, the LAP had the best discriminatory power to detect IR (area under the curve (AUC) = 0.79) (Table 2 and Figure 3). The highest Youden index for LAP corresponds to a value of 42.5, with a sensitivity of 86% and a specificity of 63%. The VAI, TG/HDL-C ratio, and TG also had good discriminatory power (AUC of 0.780.79 and 0.75, respectively) (Table 2 and Figure 3). A value of VAI of 1.44 had 86% sensitivity and 65.8% specificity, whilst a triglyceride level of 1.33 mmol/L had a sensitivity of 76.2% and a specificity of 63.7%.

**Figure 4:**
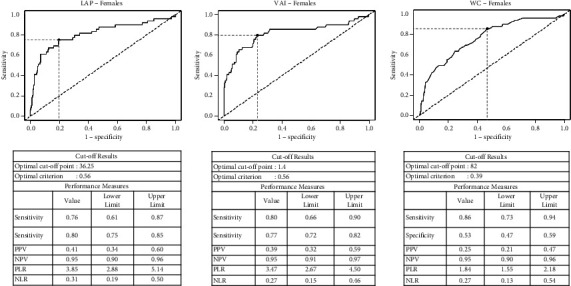
Receiver operator characteristic curves for lipid accumulation index, visceral adiposity index, and waist circumference to predict insulin resistance (HOMA-IR > 2.5) in females.

In females, VAI, LAP, and the TG/HDL-C ratio had equivalent discriminatory power to detect IR (AUC of 0.82 for VAI and TG: HDL-C ratio and 0.81 for LAP) (Table 2 and Figure 4). A value of LAP of 36.2 had a sensitivity of 75.5% and a specificity of 80.4% to detect IR, a value of VAI of 1.41 had 79.6% sensitivity and 77.8% specificity, whilst a TG/HDL-C ratio of 0.78 had a sensitivity of 77.6% and a specificity of 76.9%. TG also had good discriminatory power (AUC = 0.78), with a value of triglyceride level of 1.35 mmol/L having a sensitivity of 65.3% and a specificity of 85.9%.

Of the anthropometric parameters in females, the WC had the best discriminatory power (AUC of 0.76), followed closely by BMI (AUC 0.74) (Table 1). The optimal cutoff for WC to predict insulin resistance in females was 82 cm with a sensitivity of 85.7% and a specificity of 53.3%. The optimal cutoff for BMI in females was to 31.9 kg/m^2^, with a sensitivity of 59% and a specificity of 80%.

In males, BMI (AUC = 0.73) and waist circumference (AUC = 0.70) were the strongest anthropometric predictors of insulin resistance (Table 2). The optimal cutoff for WC to predict insulin resistance in males was 96.5 cm with a sensitivity of 72.1% and a specificity of 60.3%, while the optimal cut-off for BMI in males was 29.1 kg/m^2^, with a sensitivity of 74.4% and a specificity of 64.4%. Waist-hip ratio, BAI, AVI, FI, HDL-C, serum uric acid, liver transaminase, and weight-adjusted thigh circumference all had poor discriminatory power, whereas ferritin levels, systolic and diastolic blood pressure and “A” body shape did not exceed significance thresholds in ROC analysis.

## 4. Discussion

The lipid accumulation product (LAP), which incorporates both the WC and TG in its calculation, exhibited the highest discriminatory power in males and also performed very well in females. We also found that both the WC and TG individually had good discriminatory power in both sexes. The WC is a well-established marker of central adiposity, which in turn is strongly associated with IR. Although it also measures abdominal subcutaneous fat, which is believed to be less unhealthy than visceral fat, we found that WC is a strong predictor of IR in both sexes. It performed better than the BMI, which is consistent with previous data [38]. WC has also been shown to predict incident type 2 diabetes [39, 40] and cardiovascular disease independently of BMI [41, 42].

TG levels have been shown to exhibit a strong independent association with IR [43] and with type 2 diabetes [44, 45]. Insulin stimulates lipoprotein lipase activity; IR therefore results in reduced lipoprotein lipase activity [46–48], leading to increased triglyceride levels. Since circulating non-esterified fatty acids (also known as free fatty acids) are a major determinant of hepatic triglyceride production and packaging into very low density lipoprotein [49–51], serum triglyceride levels may be marker of free fatty acid levels. The latter are thought to be causally related to insulin resistance [52, 53]. They also inhibit lipoprotein lipase [54], resulting in a further increase in circulating triglyceride levels.

HDL-C had poor discriminatory power in both men and women, and TG/HDL-C ratio was not significantly better than TG on its own. HDL-C exhibits a higher heritability than other lipids [55]. It also has much higher heritability when compared to IR [56], implying that environmental factors that affect IR have much less impact on HDL-C. Furthermore, many of the genetic polymorphisms that have been shown to affect HDL-C concentrations would not be expected to affect insulin resistance [57–60]. It should also be noted that, although epidemiological data show that low HDL-C is associated with increased cardiovascular disease, most known genetic variants that affect HDL-C levels do not increase cardiovascular disease risk [57, 61, 62]. Dysfunctional HDL may be more important in identifying insulin resistance [63–65], but this is not captured by measuring HDL-C levels.

We found optimal cutoffs for WC in both males and females to be lower than those currently in use. In fact the optimal cut-offs were 96.5 cm in males and 82 cm in females. The National Cholesterol Education Program/Adult Treatment Panel III (NECP) recommends a cutoff of 102 cm in males and 88 cm in females in its definition of metabolic syndrome [66]. Meig's et al. [67] and Hamer and Stamatakis [68] use the same cutoffs in their definition of metabolic health. These NCEP cutoffs were developed over 20 years ago. There is evidence of secular changes in body fat distribution [16, 17] and a decrease in muscle mass [18, 19]. This might have contributed to IR occurring at a lower WC than previously. Furthermore, a decline in serum testosterone levels has been reported in males [69, 70]. Since low androgen levels are associated with a greater increase in visceral fat area compared to subcutaneous fat area [71], this may also have contributed to IR occurring at a smaller WC.

We found that in our contemporary cohort, the optimal cutoff for TG to predict insulin resistance was 1.35 mmol/L in males and 1.33 mmol/L in females, which is much lower than the 1.7 mmol/L recommended by NCEP-ATPIII [66] and many others. There are surprisingly little data to support the use of 1.7 mmol/L cutoff. Indeed, there is evidence that cardiovascular disease risk starts to increase at much lower levels. For example, a triglyceride level >0.68 mmol/l was found to predict incident cardiovascular disease risk in Korean subjects [72]. The best cutoff for nonfasting triglycerides to predict ischaemic heart disease in Japanese subjects has recently been reported to be 1.24 mmol/L [73].

### 4.1. Strengths and Limitations

We studied a reasonably-sized, well-characterized, representative cohort of middle-aged individuals. The decrease in muscle mass and function and the changes in fat distribution with ageing make it likely that the discriminatory power of the various parameters and their respective optimal cutoffs are different in elderly individuals. We therefore believe that it is important to study different age groups separately. We used standard procedures in measuring anthropometric parameters, whilst biochemical parameters were analysed in a single laboratory with appropriate quality controls.

Our study was a cross-sectional one, and it is therefore not possible to make any conclusions on intermediate or long-term outcomes. Although the euglycaemic clamp is usually considered to be the gold standard measure of IR, there is a very good correlation with HOMA-IR [10, 74]. Furthermore, values of HOMA-IR ≥ 2.5 as we used in our study have been reported to predict with increased mortality [20, 21].

We studied Maltese Caucasians since all other racial groups are underrepresented in our population. Our results therefore need to be replicated in other subpopulations.

## 5. Conclusions

Our data show that in a Maltese Caucasian middle-aged population, both the LAP and VAI exhibited good discriminatory power to detect IR (defined as HOMA-IR ≥ 2.5) in both sexes. The optimal cutoffs in males were 47.4 and 1.64, respectively, whilst in females the optimal cutoffs were 36.1 and 1.42, respectively. TG and WC also had good discriminatory power in both sexes, but with lower cutoffs than those currently recommended by NCEP-ATPIII. In fact, the optimal cutoffs for TG were 1.35 mmol/L in males and 1.33 mmol/L in females, whilst those for WC were 96.5 cm in males and 82 cm females. Our results therefore suggest that current cutoffs need to be revised downwards in this population, and future longitudinal studies are required to investigate further their relationship with hard outcomes such as type 2 diabetes, cardiovascular disease, and mortality.

## Figures and Tables

**Table 1:** Patient characteristics by gender and HOMA-IR ≥ 2.5.

	Males (*n* = 190)			Females (*n* = 331)		
	HOMA-IR < 2.5	HOMA-IR ≥ 2.5	Statistical significance	HOMA-IR < 2.5	HOMA-IR ≥ 2.5	Statistical significance
Age (years)^1^	41.3 ± 4.0	41.7 ± 3.8	*p*=0.50	40.5 ± 4.0	41.3 ± 3.8	*p*=0.11
Systolic blood pressure (mmHg)^2^	120 (115–125)	120 (115–130)	*p*=0.50	120 (115–125)	120 (115–127)	*p*=0.67
Diastolic blood pressure (mmHg)^2^	80 (80–85)	80 (80–90)	*p*=0.16	80 (75–88)	80 (70–80)	*p*=0.30
Total cholesterol (mmol/L)^2^	4.94 (4.38–5.61)	5.09 (4.47–5.98)	*p*=0.79	4.65 (4.24–5.27)	5.04 (4.41–5.55)	*p*=0.50
LDL-C (mmol/L)^2^	3.01 (2.25–3.53)	3.71 (2.54–3.71)	0.67	2.69 (2.15–3.28)	3.07 (2.50–3.48)	*p*=0.01
HDL-C (mmol/L)^2^	1.31 (1.13–1.53)	1.03 (096–1.18)	*p* < 0.001	1.59 (1.36–1.85)	1.25 (1.09–1.53)	*p* < 0.001
Triglycerides (mmol/L)	1.15 (0.84–1.57)	1.91 (1.19–2.58)	*p* < 0.001	0.85 (0.65–1.12)	1.42 (0.88–1.91)	*p* < 0.001
FPG (mmol/L)^2^	5.23 ± 4.90–5.55	5.96 (5.32–7.05)	*p*=0.003	5.01 (4.76–5.27)	5.40 (4.90–5.98)	*p* < 0.001
BMI (kg/m^2^)^2^	27.5 (24.1–36.9)	33.4 (29.4–36.9)	*p* < 0.001	25.6 (23.1–28.7)	34.5 (31.8–39.2)	*p* < 0.001
Waist circumference (cm)^2^	93.0 (87.0–101.4)	102.5 (94.3–110.0)	*p* < 0.001	81 (71.6–91.5)	94 (88.8–107)	*p* < 0.001
On lipid-lowering medication^3^	5 (3.4%)	3 (7.0%)	*p*=0.30	6 (2.1%)	11 (22.0%)	*p* < 0.001
On antihypertensive medication^3^	7 (4.8%)	7 (16.3%)	*p*=0.01	5 (1.8%)	13 (26.0%)	*p* < 0.001

^1^Data are mean ± standard deviation; ^2^Data are median (interquartile range); ^3^Data are number (percentage). BMI = body mass index; FPG = fasting plasma glucose; HDL-C = high-density lipoprotein cholesterol; HOMA-IR = homeostasis model assessment for insulin resistance; LDL-C = low-density lipoprotein cholesterol.

**Table 2:** Area under the curve of receiver operator characteristics curves for various anthropometric parameters for predicting HOMA-IR ≥ 2.5, stratified by sex.

Parameter	Males (*n* = 129)					Females (*n* = 328)				
	AUC	SE	*p* value	LCI 95%	UCI 95%	AUC	SE	*p* value	LCI 95%	UCI 95%
Body mass index (kg/m^2^)	0.73	0.04	<0.01	0.65	0.81	0.74	0.04	<0.01	0.67	0.82
Waist circumference (cm)	0.70	0.05	<0.01	0.61	0.79	0.76	0.04	<0.01	0.69	0.84
Hip circumference (cm)	0.68	0.05	<0.01	0.59	0.77	0.71	0.04	<0.01	0.63	0.79
Neck circumference (cm)	0.66	0.05	<0.01	0.57	0.75	0.71	0.04	<0.01	0.63	0.79
Average thigh circumference (cm)	0.52	0.05	0.65	0.42	0.63	0.68	0.04	<0.01	0.59	0.76
Average arm circumference (cm)	0.62	0.05	0.02	0.53	0.72	0.73	0.04	<0.01	0.65	0.81
Waist: height ratio	0.72	0.05	<0.01	0.63	0.81	0.76	0.04	<0.01	0.70	0.84
Waist: thigh ratio	0.71	0.04	<0.01	0.63	0.80	0.66	0.04	<0.01	0.58	0.75
Weight-adjusted thigh circumference (cm)	0.26	0.04	<0.01	0.18	0.34	0.33	0.05	<0.01	0.24	0.41
Waist: hip ratio	0.65	0.05	<0.012	0.56	0.75	0.67	0.04	<0.01	0.59	0.75
Visceral adiposity index	0.78	0.04	<0.01	0.70	0.86	0.82	0.04	<0.01	0.73	0.90
Lipid accumulation product	0.79	0.04	<0.01	0.71	0.87	0.81	0.04	<0.01	0.73	0.89
Body adiposity index	0.69	0.05	<0.01	0.59	0.78	0.72	0.04	<0.01	0.65	0.80
Abdominal volume index	0.70	0.05	<0.01	0.61	0.79	0.76	0.04	<0.01	0.68	0.83
Fat index	0.63	0.05	<0.01	0.53	0.73	0.70	0.04	<0.01	0.62	0.78
Triglycerides (mmol/l)	0.74	0.05	<0.01	0.66	0.83	0.77	0.04	<0.01	0.68	0.86
HDL-C (mmol/l)	0.23	0.04	<0.01	0.15	0.31	0.20	0.04	<0.01	0.13	0.27
TG: HDL-C ratio	0.78	0.04	<0.01	0.70	0.86	0.82	0.04	<0.01	0.74	0.90
Uric acid	0.57	0.05	0.20	0.47	0.66	0.63	0.04	<0.01	0.54	0.71
ALP	0.57	0.05	0.16	0.47	0.68	0.68	0.04	<0.01	0.60	0.76
GGT	0.62	0.05	0.02	0.53	0.71	0.69	0.04	<0.01	0.62	0.77
ALT	0.54	0.05	0.44	0.44	0.64	0.63	0.04	<0.01	0.55	0.72

ALP = alkaline phosphatase; ALT = alanine transaminase; GGT = *γ* glutamyl tansferase; HDL-C = high-density lipoprotein cholesterol.

## Data Availability

The data used to support the findings of this study are available from the authors upon reasonable request.
